# Targeting NRF2, Regulator of Antioxidant System, to Sensitize Glioblastoma Neurosphere Cells to Radiation-Induced Oxidative Stress

**DOI:** 10.1155/2020/2534643

**Published:** 2020-06-15

**Authors:** Paulo R. D. V. Godoy, Ali Pour Khavari, Marzia Rizzo, Elza T. Sakamoto-Hojo, Siamak Haghdoost

**Affiliations:** ^1^Department of Molecular Biosciences, The Wenner-Gren Institute, Stockholm University, Svante Arrhenius Väg 20C, Zip Code: 106 91 Stockholm, Sweden; ^2^Department of Biology, Faculty of Philosophy, Sciences and Letters at Ribeirão Preto, University of São Paulo, Av. Bandeirantes 3900, Zip Code: 14040-901 Ribeirão Preto, SP, Brazil; ^3^Department of Genetics, Faculty of Medicine of Ribeirão Preto, Av. Bandeirantes 3900, Zip Code: 14049-900 Ribeirão Preto, SP, Brazil; ^4^University of Caen Normandy, UMR6252 CIMAP/LARIA team, Zip Code: 14076 Caen, France; ^5^Advanced Resource Center for HADrontherapy in Europe (ARCHADE), Zip Code: 14000 Caen, France

## Abstract

The presence of glioma stem cells (GSCs), which are enriched in neurospheres, may be connected to the radioresistance of glioblastoma (GBM) due to their enhanced antioxidant defense and elevated DNA repair capacity. The aim was to evaluate the responses to different radiation qualities and to reduce radioresistance of U87MG cells, a GBM cell line. U87MG cells were cultured in a 3D model and irradiated with low (24 mGy/h) and high (0.39 Gy/min) dose rates of low LET gamma and high LET carbon ions (1-2 Gy/min). Thereafter, expression of proteins related to oxidative stress response, extracellular 8-oxo-dG, and neurospheres were determined. LD50 for carbon ions was significantly lower compared to LD50 of high and low dose rate gamma radiation. A significantly higher level of 8-oxo-dG was detected in the media of cells exposed to a low dose rate as compared to a high dose rate of gamma or carbon ions. A downregulation of oxidative stress proteins was also observed (NRF2, hMTH1, and SOD1). The NRF2 gene was knocked down by CRISPR/Cas9 in neurosphere cells, resulting in less self-renewal, more differentiated cells, and less proliferation capacity after irradiation with low and high dose rate gamma rays. Overall, U87MG glioma neurospheres presented differential responses to distinct radiation qualities and NRF2 plays an important role in cellular sensitivity to radiation.

## 1. Introduction

Glioblastoma (GBM) is the most common type of malignant brain tumor in adults reaching 3.6 cases per 100,000 persons per year in Europe [1]. Survival of GBM patients is around 12–15 months after diagnosis, even after surgical resection, chemo-, and radiotherapy [2]. Genetic heterogeneity is characteristic of GBM [3]. The poor prognosis for GBM patients is due to the GBM resistance to chemotherapy and ionizing radiation [4], which may be linked to cancer stem cells (CSCs) in the tumor mass [5–7]. The resistance ability of CSCs appears to be associated with their slow-cycling phenotype, and/or expression of efflux transporters, antiapoptotic proteins, altered profile of cell surface markers, effective DNA damage response and repair mechanisms, or the presence of elevated free radical scavengers (reviewed in [8]).

Considering that it is an extremely difficult task to study CSCs isolated from primary tumors, it was shown that even after years of culturing under differentiating conditions, glioblastoma cell lines contained a fraction of cells able to form neurospheres when cultured under stem cell conditions (*in vitro*) [9–12]. There is evidence that GBM tumors may originate from CSCs. CSCs can grow as neurospheres and often reflect the histopathological features of the tumor, indicating their suitability to reproduce the cellular heterogeneity of human GBM (13), which would help to find molecular therapeutic targets related to CSCs. The expression of stem cell markers is increased in neurospheres compared to their corresponding adherent cells [12] showing clonogenicity, long-term renewal capacities and multilineage differentiation. However, not only CSCs but also their transit-amplifying progeny are able to form spheres [13, 14].

It has already been described that higher levels of reactive oxygen species (ROS) in tumors compared to normal cells are related to various mechanisms, e.g., mitochondrial dysfunction, peroxisome activity, oncogene activity, less effective antioxidant system, and glucose metabolism [15, 16]. A more efficient antioxidant defense mechanism was found in cultures enriched for glioma stem cells (GSCs), cultured in serum-free medium supplemented with growth factors, when comparing them with differentiated counterparts after treatment with hydrogen peroxide [17]. This may be due to the higher activated mitochondrial metabolism and production of ATP when comparing GSCs and their nontumorigenic tumor-cell counterparts, which leads to higher ROS levels, demanding high levels of antioxidant mechanisms [18]. Targeting a component of the antioxidant system or increasing ROS generation seems to be promising strategies to decrease GBM cell viability. When GSCs were treated with a glutathione (GSH) inhibitor, buthionine sulfoximine (BSO), an accumulation of intracellular ROS and induction of differentiation were observed [11]. It was also shown that peroxiredoxin 4 (PRDX4) knockdown by shRNA increased the ROS formation and unfolded protein levels, apoptosis induction, and decreased growth of GSCs *in vitro* and *in vivo* [19]. Other authors described that the interference in the mitochondrial respiration through TRAP1 and Sirtuin-3 modulation caused an increase in ROS generation, leading to metabolic alterations, loss of stemness, and suppression of tumor formation *in vivo* [20]. However, recent studies reported that cells expressing CSC-associated cell membrane markers in GBM do not represent a clonal entity defined by distinct functional properties and transcriptomic profiles, but rather a plastic state that most cancer cells can adopt. The capacity of any given cancer cell to reconstitute tumor heterogeneity seems to be a restriction against therapies targeting CSC-associated membrane epitopes [21].

The role of ROS in the GBM microenvironment, including GSCs, still needs better characterization [22], particularly in response to different types of radiation with different LET. ROS can be generated by ionizing radiation, which could lead to base alterations, single-strand breaks (SSBs), oxidative base damage, and double-strand breaks (DSBs) [23, 24]. Hadrontherapy, particle radiation therapy, has been suggested to be an approach to overcome GBM CSCs. In particular, when compared with photons, charged particles seem to be more effective in CSCs' killing due to different qualities of induced DNA damage [25]. Particle irradiation induces a higher amount of multiple DNA damage sites (MDS) as compared with low LET radiation. In addition to DSBs, particle irradiation can induce non-DSB oxidative clustered DNA lesions (OCDL), including oxidized bases and apurinic-apyrimidinic (abasic, AP) sites [26, 27]. Exposure to particle radiation was found to induce persistent oxidative stress in mouse intestine cells, indicating that the oxidative stress is an important factor after this type of radiation [28]. Proton radiation, compared to photons, is more effective in killing the exposed GSCs due to the production of more complex DNA damage and ROS [29]. Here, we studied different radiation qualities, low and high dose-rate gamma irradiation, and carbon ions. These three radiation qualities kill cells by induction of slightly different DNA damage qualities and different relative biological effectiveness factors. While carbon ion irradiation results in very cytotoxic MDS along its traverse in DNA, high dose rate exposure to gamma irradiation produces randomly distributed DNA damage within a short time; in contrast, low dose rate irradiation (mGy/h range) induces DNA damage over a long period of time, providing time to efficiently repair the DNA damage.

The nuclear factor erythroid 2- (NFE2-) related factor 2 (NRF2) is considered a master regulator of oxidative stress responses. During unstressed condition, NRF2 is bound to KEAP1, being subsequently degraded following ubiquitination. After cellular exposure to chemical toxins and radiation, NRF2 is dissociated from KEAP1, accumulates in the nucleus, and activates several genes related to detoxification and antioxidant response, protecting cells from DNA damage induction [30–33]. NRF2 controls the expression of several proteins that contribute to GSH homeostasis [34] and superoxide dismutase (SOD) 1 [35, 36]. SOD is important for the dismutation of superoxide anion radicals into H_2_O_2_ and oxygen molecules [37].

Acting upstream of NRF2, SGK1 induces NRF2 expression and antioxidant activation in a c-JUN-dependent manner [38]. Furthermore, the PI3K and AKT functions are also required for NRF2 activation [39–44]. Upregulation of the PI3K/AKT pathway has also been documented in GSCs, thus promoting self-renewal and tumor formation [45].

NRF2 downregulation or inhibition decreases GBM proliferation or promotes sensitization to temozolomide, a chemotherapy drug, and gamma radiation [46–48]. Inhibition of SGK1, upstream of NRF2, by SI113, a drug designed *in silico* [49], induced cell death, altered growth rate as well as autophagy, and modulated the response to oxidative stress in human GBM cells [50]. It was also shown that SGK1 inhibition reduces radioresistance of cervical cancer [38] and synergizes with autophagy inhibitors and ^64^CuCl_2_ effects in GBM cells [51, 52]. Besides, SGK1 inhibition has little effect on traditional serum-grown glioma lines and on differentiated GSCs, which has been shown to be important for GBM stem cells [53].

However, NRF2 inhibition was never tested in GBM neurospheres treated with low or high dose rates of gamma radiation. Our hypothesis is that reducing the antioxidant capacity of neurospheres by reducing NRF2 expression and increasing ROS production by low and high dose-rate irradiation will reduce the GSCs' functionality, increasing differentiation and decreasing self-renewal. In the present study, we aimed to evaluate the GBM neurospheres regarding molecular and cellular responses to different doses of gamma radiation delivered at low or high dose rates, as well as carbon ion radiation. The second aim was to establish GBM neurospheres containing stem cells presenting reduced NRF2 expression and also to evaluate cellular radiosensitivity to gamma irradiation delivered at different dose rates (low and high).

## 2. Materials and Methods

### 2.1. Cell Lines, Cell Culture, and Irradiation

The following GBM cell lines were purchased from the American Type Culture Collection (ATCC, Manassas, VA, USA): U87MG (HTB-14), T98G (CRL-1690), LN18 (CRL-2610), M059K (CRL-2365), and M059J (CRL-2366). The cells were cultured in DMEM/F12 medium (Sigma-Aldrich, St. Louis, MO, USA), supplemented with 10% fetal bovine serum (HyClone, Thermo Fisher Scientific, Waltham, MA, USA) and 1% PenStrept (10.000 U penicillin and 10 mg streptomycin/ml, Sigma-Aldrich). There has been some uncertainty regarding the origin of the U87MG (HTB-14) glioma cell line from ATCC [54]; however, this does not interfere with the results of the present study. The U87MG from ATCC is a confirmed GBM cell line. Our group authenticated U87MG (by STR profiling method) [55]. It has even its genome decoded [56] and has been used in most of the recent studies on GBM. The majority of the available comparative research articles which also have been cited in the present investigation are based on the U87MG cell line from ATCC.

Glioma stem cells (GSCs) from U87MG were enriched by culturing U87MG in noncoated flasks with neural stem cell medium (NSCM), leading to the formation of nonadherent cell aggregates referred to as neurospheres. The NSCM consists of DMEM/F12 serum-free medium (Sigma-Aldrich, St. Louis, MO, USA) supplemented with B27 without antioxidants (Invitrogen; Thermo Fisher Scientific, Inc., Waltham, MA, USA), 20 ng/ml EGF (PeproTech EC Ltd., London, UK), 20 ng/ml FGF2 (PeproTech EC Ltd. London, UK), and 1% PenStrept (Sigma-Aldrich, St. Louis, MO, USA). Under this culture condition, neurospheres were formed, dissociated with Accutase (Sigma-Aldrich, St. Louis, MO, USA) and reseeded at 3- to 4-day intervals. After 3 rounds of this passaging procedure, neurospheres were dissociated, counted, and frozen (10% DMSO, Sigma-Aldrich, St. Louis, MO, USA) in different vials to be used for the start of each unique experiment. Differentiated cells were generated after cultivating these neurosphere cells in DMEM/F12 (Sigma-Aldrich, St. Louis, MO, USA), supplemented with 10% FBS (HyClone, Thermo Fisher Scientific, New Zealand), and incubated at 37°C with 5% CO_2_ for at least 1 week, where they grow attached to the flask. Only LN18 was cultured in regular DMEM instead of DMEM/F12.

### 2.2. Irradiation Process

U87MG, T98G, LN18, M059K, and M059J cells were cultured as monolayer in triplicate in 96-well plates. After 24 hours of incubation at 37°C, the plates were exposed to 0, 0.5, 2, 4, and 8 Gy of gamma radiation (Scanditronix 0.39 Gy/min, ^137^Cs). After irradiation, the cells were kept at 37°C in 5% CO_2_ for 7 days. The cell viability was determined by adding 0.1 mg/mL resazurin (Sigma-Aldrich) in the culture medium (phenol-free DMEM, supplemented with 10% FBS, Sigma-Aldrich) followed by incubation for 1 hour at 37°C. The signal detection from resazurin was determined using fluorescence detector (POLARstar Omega Plate Reader, BMG LabTech, Ortenberg, Germany) measured at 535/590 nm.

The radiosensitivity of cells was established, and the U87MG cell line was selected for the planned experiments in the present investigation because it presented one of the highest LD50s and a high capacity of forming neurospheres.

**Figure 1:**
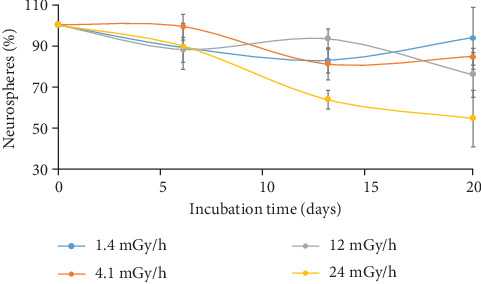
Neurosphere formation analyzed after 6, 13, and 20 days in cells irradiated with different low dose rates. Cells were counted and reseeded each week.

Low dose rate irradiation was performed in a cell culture incubator positioned over a ^137^Cs source. The dose rate could be changed by lead filters and changing the distance from the radiation source. Nonexposed cells were cultured in a separate incubator. The dose rates were measured using ionization chamber TM 30010, PTW, (Freiburg, Germany) connected to Unidos E (Freiburg, Germany) equipment. Low dose rate irradiation was carried out continuously, except during the periods of subculturing. Initially, we cultured neurospheres at different low dose rates to check the cell growth over time. The following dose rates were used: 1.4, 4.1, 12, and 24 mGy/h. We found that among the studied dose rates, 24 mGy/h resulted in a significant reduction of the amount of neurospheres after 2 and 3 weeks of continuous irradiation (Figure 1). The dose rate of 24 mGy/h was chosen to be used in the present study.

Five hundred cells dissociated from neurospheres were seeded per well, in replicates of 5 wells (96-well plates), or 10.000 cells in 25 cm^2^ flasks in NSCM. After 24 hours, cells were incubated in a culture incubator containing a ^137^Cs source (24 mGy/h). Every 3 days, we added additional NSCM to the 96-well plates and flasks. After 6 days, the formed neurospheres in the 96-well plates were counted and the neurospheres from the flasks were dissociated into single cells with Accutase and counted in the Countess® Automated Cell Counter (Invitrogen) using Trypan blue (Invitrogen). These single cells from control and irradiated groups were reseeded in 96-well plates and flasks for the coming week, so the cells that were inside the radiation incubator would accumulate higher doses according to the total incubation time. This process was repeated 2 more times during 3 weeks. The total doses applied to cells irradiated at 24 mGy/h were 3.5 (6 days), 7.5 (13 days), and 11.5 Gy (20 days), respectively. For each subculture time, we kept the medium for the detection of 8-oxo-dG and cell pellets for protein extraction.

**Figure 2:**
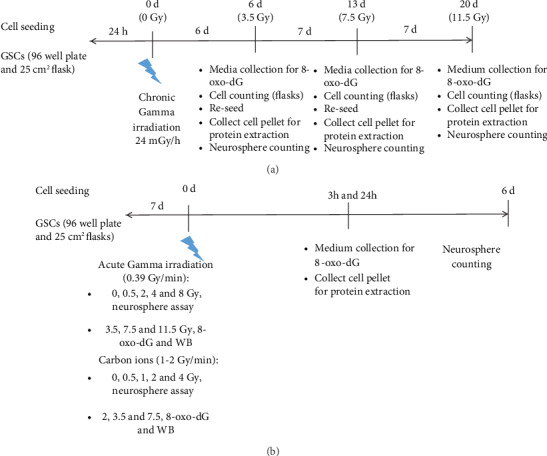
Fluxogram of the experiment setup for neurospheres irradiated with low dose rate (a) and high dose rate gamma radiation and carbon ions (b).

In parallel, we also analyzed the responses of neurospheres to high dose rate, low LET radiation, and carbon ions. Similar experimental and cell culture conditions were used during high dose rate irradiation by gamma and carbon ions. For high dose rate of gamma irradiation (^137^Cs 0.39 Gy/min, Scanditronix, Uppsala, Sweden), cellular responses to 3.5, 7.5, and 11.5 Gy were analyzed for protein expression at 3 and 24 hours post irradiation, and analyses of 8-oxo-dG in the medium were performed 3 h after irradiation. The following doses were used for the analysis of sphere formation: 1, 2, 4, and 8 Gy, Figure 2(b).

Neurosphere cells were exposed to ^12^C beam irradiation (1-2 Gy/min, 97 MeV/*μ*m LET) at the GANIL facility (Caen, France). The doses were 0.5, 1, 2, and 4 Gy for sphere formation determined at 6 days after exposure, and doses of 2, 3.5, and 7.5 Gy for the experiments involving protein analysis and 8-oxo-dG detection, performed in cells collected at 3 and 24 hours after exposure, Figure 2(b). Similar doses were used to compare the effects of each radiation quality. However, as carbon ions are known to be more cytotoxic to GSCs than gamma [18], we used lower doses of carbon ions for all endpoints. Since the exposure of cells to low dose rate for 2-3 weeks caused low cell proliferation, it was not possible to collect an adequate number of cells to perform protein analysis at 24 hours post irradiation.

### 2.3. Sphere Formation Assay

We seeded 500 cells/well (cell concentration was chosen based on previous tests with 250, 500 and 1000 cells/well) in noncoated 96-well plates, quintuplicate wells with 100 *μ*L of neurosphere formation medium for 7 days in total. Cells were irradiated 24 hours after seeding. Extra 100 *μ*L medium was added 3-4 days after irradiation. Neurospheres over 60 microns were counted using an inverted microscope at 100x magnification.

### 2.4. Western Blotting (WB)

Briefly, cells were lysed in standard Laemmli buffer supplemented with a proteinase inhibitor cocktail (Roche Diagnostics GmbH, Mannheim, Germany). Proteins were quantified using Pierce Protein Assay Reagent (Thermo Scientific, Rockford, USA) according to the manufacturer's instructions, and 10 *μ*g were used for WB.

Proteins were separated by electrophoresis in NuPAGE 4–12% Bis–Tris gel (Invitrogen) and blotted onto a nitrocellulose membrane (Thermo Scientific) using the XCell SureLock™ Mini-Cell System (Invitrogen), overnight at 30 V (4°C). Samples were incubated for 90 min in LI-COR blocking buffer (LI-COR, Cambridge, UK) and Tris-buffered saline containing 0.05% Tween (TBST), (1 : 1), before the incubation with primary antibody: NRF2 (SAB4501984, 1 : 500, rabbit, Sigma-Aldrich); SOD1 (4266S, 1 : 1000, mouse, Cell Signaling Technology, Inc., Danvers, MA); SOD2 (13141S, 1 : 2000, rabbit, Cell Signaling Technology, Inc.,); GSS (WH0002937M1, 1 : 1000, mouse, Sigma-Aldrich); GSTO1 (WH0009446M1, 1 : 1000, mouse, Sigma-Aldrich,); PRDX2 (R8656, 1 : 1000, rabbit, Sigma-Aldrich); hMTH1 (NB100-109, 1 : 1000, rabbit, Novus,), MUSASHI-1 (5663S, Cell Signaling Technology, Inc., rabbit, 1 : 1000), APE1 (1 : 1000, a gift from the group of professor Murat Saparbaev, France), and GAPDH (G8795, Sigma, mouse, 1 : 10000). Primary antibody was diluted in the blocking buffer, and incubation took place overnight at 4°C. Secondary antibody was anti-mouse or anti-rabbit conjugated with IRDye® Infrared Dyes (LI-COR). Thereafter, the membranes were scanned in the Odyssey imaging system. Densitometric analysis of Western Blot bands was performed using Image Studio ver 5.2 software (LI-COR).

### 2.5. GSC Differentiation

Differentiation potential was examined by culturing neurosphere cells under the differentiation-inducing culture condition (DMEM/F12 containing 10% FBS) for 7 days, followed by cell lysis for protein extraction, described before. Then, a Western Blot was performed to analyze MUSASHI-1 in the cells by anti-MUSASHI-1 (Cell Signaling Technology, Inc.). The differentiated cells grow attached to the flask, while the neurospheres grow floating.

### 2.6. Competitive ELISA for Determination of Extracellular 8-oxo-dG in the Media

The 8-oxo-dG concentration of media was measured using enzyme-linked immunosorbent assay, ELISA (Health Biomarkers Sweden AB, Stockholm, Sweden) [57]. Extracellular 8-oxo-dG (blood serum, cell culture medium) as well as the 8-oxo-dG content of DNA has been used as a marker for radiation-induced oxidative stress [58]. The technique to detect 8-oxo-dG using ELISA was developed by Hagdhoost et al. [58], the Genetic and Toxicology group, Stockholm University, and made it possible to analyze the nucleotide pool sanitization using various sources, including cell culture medium [58].

Briefly, media from the treated and untreated cells (neurospheres) were collected 3 hours after treatment and then freeze-dried overnight. The dried pellet was dissolved in cold distilled water, loaded on a C18 solid-phase-extraction column (Varian, Lake Forest, CA, USA) and washed with PBS (pH 7.4). Then, 8-oxo-dG was eluted with PBS, pH 7.4, containing 20% methanol, freeze-dried, and purified again. Samples were dissolved in PBS (pH 7.4), mixed with primary antibody against 8-oxo-dG (Japan Institute for the Control of Aging, Japan) and transferred to 96-well ELISA plates precoated with 8-oxo-dG. After overnight incubation at 4°C, the plates were washed with washing solution (PBS, pH 7.4, 0.02% Tween 20 and 0.1% bovine serum albumin) followed by addition of HRP-conjugated secondary antibody (goat anti-mouse IgG-HRP, Scandinavian Diagnostic Services, Sweden) and 2 hours of incubation at room temperature. Then, the wells were washed and incubated with tetramethylbenzidine liquid substrate (ICN Biomedicals Inc.) in the dark for 15 min. The reaction was terminated by adding 2M H_3_PO_4_ (Merck Millipore, Darmstadt, Germany). The absorbance was measured at 450 nm using an automatic ELISA plate reader. To display the levels of 8-oxo-dG as ng/million cells, the determined 8-oxo-dG level in the pooled samples (ng/ml) was multiplied with the corresponding total volume of the media collected during the experiment, divided by the total number of cells obtained.

### 2.7. CRISPR-CAS9 Gene Knockout

On day one, 10.000 U87MG neurospheres were seeded in 96-well plates containing 100 *μ*L NSCM and incubated for 24 hours. Next day, two different plasmids targeting different exons of the NRF2 gene (Target ID HS0000147047 and HS0000147051, ready-to-use Cas9 and guide RNA expression plasmids for NRF2, Sigma-Aldrich) were pooled (100 ng of each) and U87MG neurosphere cells were transfected using Lipofectamine® 3000 (Invitrogen; Thermo Fisher Scientifc, Inc.), according to the manufacturer's protocol, and incubated for 6 hours in DMEM/F12 medium. Then, complete NSCM medium was added and cells were incubated for 72 hours; next, cells were counted and seeded as single cell/well in 96-well plate. This allowed obtaining several plates with colonies formed from single transfected cells. Western Blot analysis of the target protein, NRF2, was performed on cells from several transfected clones to test the levels of knockout. We chose the clone with the lowest NRF2 expression that could grow and form spheres to be used in the experiments.

### 2.8. Statistical Analysis

In general, at least 3 independent experiments were performed. The results were analyzed by One-Way ANOVA, followed by the Holm-Sidak multicomparison test when comparing more than two groups, or Student's *t*-test, when comparing 2 groups. A *P* value < 0.05 was considered significant. Statistical tests were performed using SigmaStat for Windows Version 3.5 (Systat Software, Inc., San Jose, CA, USA), while the graph plots were performed using Excel version 2010 (Microsoft Corporation, Redmond, WA, USA). Plotted results represent the average of experiments, and bars correspond to standard deviations.

## 3. Results

### 3.1. Radioresistance of GBM Cell Lines

**Figure 3:**
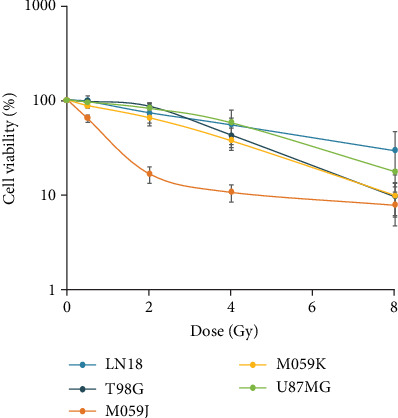
Comparison of cell viability (% of control) among five different cell lines (LN18, T98G, M059J, M059K, and U87MG) after 0.5, 2, 4, and 8 Gy gamma irradiation using resazurin fluorescence detection in a plate reader (*n* = 3).

In order to examine the sensitivity of GBM cells to radiation, we exposed five cell lines with different genetic backgrounds to gamma radiation. The cells were exposed to 0, 0.5, 2, 4, and 8 Gy at a high dose rate (0.39 Gy/min), and cell viability was analyzed using resazurin assay in 96-well plates. Cells presented different sensitivities following 120 hours post irradiation. Most of them showed similar responses, with decrease of viability as a function of radiation doses (Figure 3). However, it was noticed that M059J is the most radiosensitive cell line, probably due to the DNA PK mutation [59]. LD50 was calculated for each cell line (Table 1). U87MG cells were chosen for further investigation on the basis of their high LD50 and high potential to form spheres, compared to several other cell lines [12, 60]. Despite that the origin of U87MG has been questioned [54], it has been used in most of the recent literature, and we performed the authentication of U87MG from ATCC.

### 3.2. Stemness Properties of Cells Analyzed in U87MG Neurospheres

**Figure 4:**
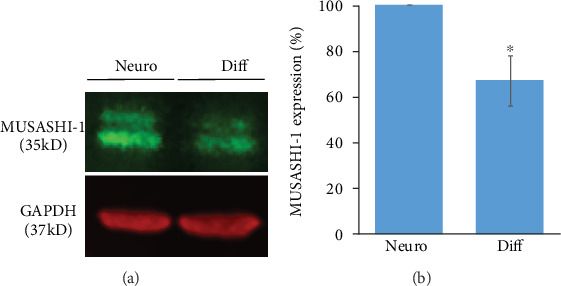
Expression of MUSASHI-1 in U87MG neurospheres and differentiated cells (a). Quantitative analyses of the WB bands were used to calculate the relative expression of MUSASHI-1 (b). ∗*P* ≤ 0.05.

Neurospheres were obtained by culturing U87MG cells in the GSCs' media. To obtain GSC enrichment, neurospheres were grown and subcultured several times prior to the experiments. Western Blot showed a significantly higher expression of MUSASHI-1 (*P* = 0.005) in neurospheres (34%), a known stem cell marker for GBM (Figure 4), compared to differentiated cells. We have already shown that the CD133+ marker was also upregulated in neurospheres of the U87MG cell line, compared to serum-differentiated cells [61]. Several other authors also showed increased expression of stem cell markers in U87MG neurospheres compared to their serum-derived counterparts [12, 62].

### 3.3. Expression of Oxidative Stress Proteins in GBM Cell Lines

**Figure 5:**
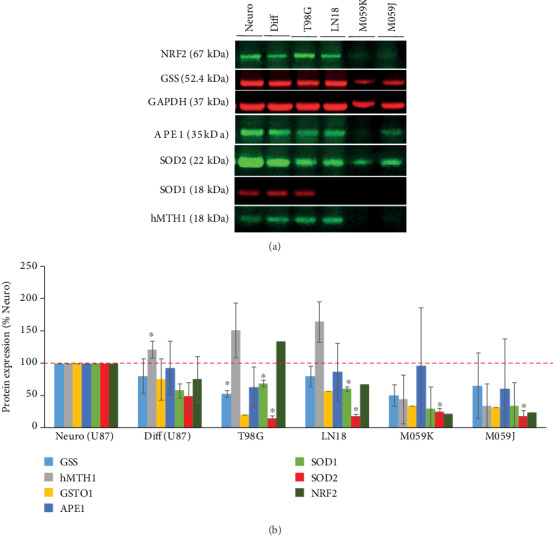
Western Blot analysis of GSS, GSTO1, hMTH1, SOD1, SOD2, APE1, and NRF2 in neurospheres (U87MG), differentiated U87MG, and T98G, LN18, M059K, and M059J GBM cell lines without any treatment (a). Quantitative analysis of the WB bands was used to calculate the relative expression of each protein normalized by GAPDH used as endogenous control. Relative expression was calculated compared to neurospheres (b), *n* = 3, ∗*P* ≤ 0.05.

We also analyzed expression of proteins involved in oxidative stress response, e.g., GSS, GSTO1, hMTH1, SOD1, SOD2, APE1, and NRF2, by comparing U87MG neurospheres, differentiated U87MG cells and 4 other GBM cell lines: T98G, LN18, M059K, and M059J. Considering the protein expression levels under nonirradiation condition, a reduced expression of stress-related proteins in differentiated U87, LN18, T98G, M059K, and M059J was observed in comparison with U87 neurospheres (Figures 5(a) and 5(b)).

The results showed a trend of downregulation of GSS, APE1, SOD1, SOD2, and NRF2 in almost all cell lines when compared to neurospheres (Figures 5(a) and 5(b)). Significant reduction of the expression levels were observed for the following proteins: GSS (T98G, *P* = 0.006), SOD1 (T98G, *P* = 0.014; LN18, *P* = 0.004), and SOD2 (T98G, *P* = 0.001; LN18, *P* ≤ 0.001; M059K, *P* = 0.003; M059J, *P* = 0.006). On the opposite, a significant increase was observed for hMTH1 expression in differentiated U87 (*P* = 0.041) compared to hMTH1 expression in neurospheres (Figure 5(b)).

### 3.4. Comparison Between Different Radiation Qualities on Neurospheres' Formation

**Figure 6:**
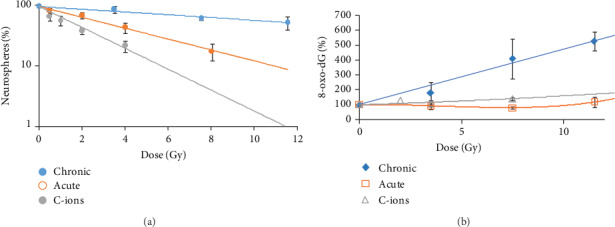
(a) Neurosphere formation after low and high dose rate gamma and carbon ion radiation. (b) The levels of 8-oxo-dG in the media after low and high dose rates, low LET gamma radiation, and high LET carbon ions. The values are relative to sham-irradiated control, *n* = 3.

We compared the formation of neurospheres following treatment with 3 different radiation qualities: low and high dose rates of low LET gamma and high LET carbon ion radiation. All the three radiation qualities reduced the neurosphere formation. LD50 values of low dose rate gamma, high dose rate gamma, and carbon ions were 11.1 ± 3.1 Gy, 3.5 ± 0.6 Gy, and 1.3 ± 0.4 Gy, respectively. Significant differences were found by comparing LD50 for low versus high dose rate (*P* = 0.010), low dose rate (gamma) versus carbon ions (*P* ≤ 0.001) and carbon ions versus high dose rate (gamma) irradiation (*P* = 0.003) (Figure 6(a)).

Extracellular 8-oxo-dG (in urine, blood serum, and cell culture media) can be formed after oxidative stress, and it has been considered a sensitive marker of oxidative stress. Low dose rate irradiation significantly increased the generation of 8-oxo-dG following 3.5, 7.5, and 11.5 Gy in a dose-dependent manner (Figure 6(b)). Higher levels of 8-oxo-dG were found after low dose rate irradiation compared to high dose rate (*P* = 0.009, *P* = 0.012, and *P* ≤ 0.001 for 3.5, 7.5, and 11.5 Gy, respectively) and carbon ions (*P* = 0.018 and *P* ≤ 0.001 for 3.5 Gy and 7.5 Gy, respectively). A nonsignificant increase was observed in 8-oxo-dG levels presented by carbon ion-irradiated cells in comparison with cells irradiated with high dose-rate (Figure 6(b)).

**Figure 7:**
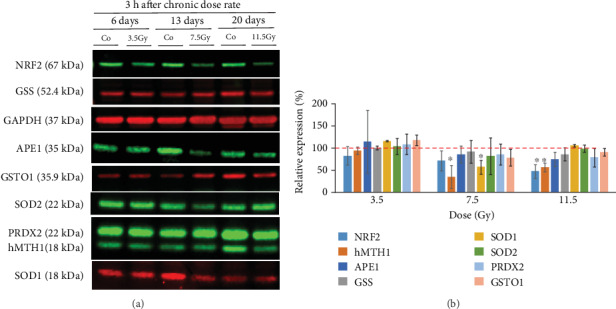
Western Blot analysis of GSS, GSTO1, hMTH1, SOD1, SOD2, PRDX2, APE1, and NRF2 in neurosphere cells irradiated with 3.5, 7.5, and 11.5 Gy of gamma (low dose rate) (a). Quantitative analysis of the WB bands was used to calculate the relative expression of each protein normalized by GAPDH (b). ∗*P* ≤ 0.05.

We also investigated the expression levels of stress-related proteins in neurosphere cells analyzed at a low dose rate. The results showed that low dose rate irradiation reduced the expression of several stress-related proteins, particularly at 7.5 (6 days of exposure) and 11.5 Gy (20 days of exposure) (Figures 7(a) and 7(b)). We observed a significant reduction in hMTH1 (*P* = 0.001) and SOD1 (*P* = 0.005) expression for 3.5 Gy and also for NRF2 (*P* = 0.004) and hMTH1 (*P* = 0.007) expression for 11.5 Gy.

**Figure 8:**
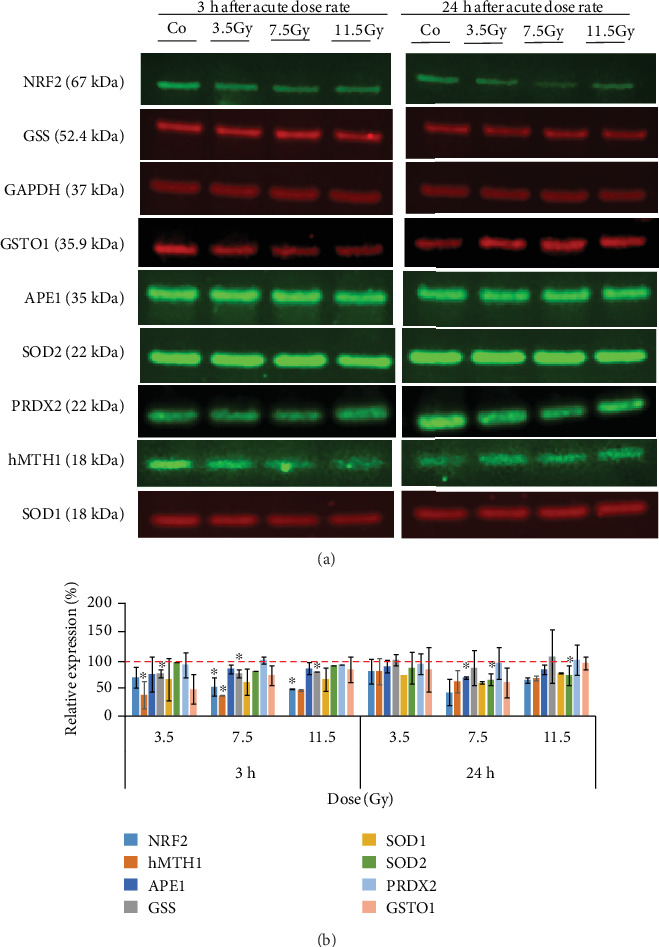
Western Blot analysis of GSS, GSTO1, hMTH1, SOD1, SOD2, PRDX2, APE1, and NRF2 in neurosphere cells irradiated with 3.5, 7.5, and 11.5 Gy of high dose-rate exposure to gamma (a). Quantitative analysis of the WB bands was used to calculate the relative expression of each protein normalized by GAPDH (b). ∗*P* ≤ 0.05, *n* = 3.

The results showed a clear trend of downregulation of several proteins in neurosphere cells at 3 hours after high-dose gamma radiation. A significant decrease was found for NRF2 expression after irradiation with 7.5 (*P* = 0.02) and 11.5 Gy (*P* = 0.01), for hMTH1, 3.5 (*P* = 0.006), 7.5 (*P* = 0.006), and 11.5 Gy (*P* = 0.010) and for GSS analyzed after 3.5 (*P* = 0.0069), 7.5 (*P* = 0.008), and 11.5 Gy (*P* = 0.01). Interestingly, at the time point of 24 hours post irradiation, APE1 (7.5 Gy; *P* = 0.012) and SOD2 (7.5 Gy; *P* = 0.006 and 11.5 Gy; *P* = 0.02) were significantly downregulated (Figures 8(a) and 8(b)).

**Figure 9:**
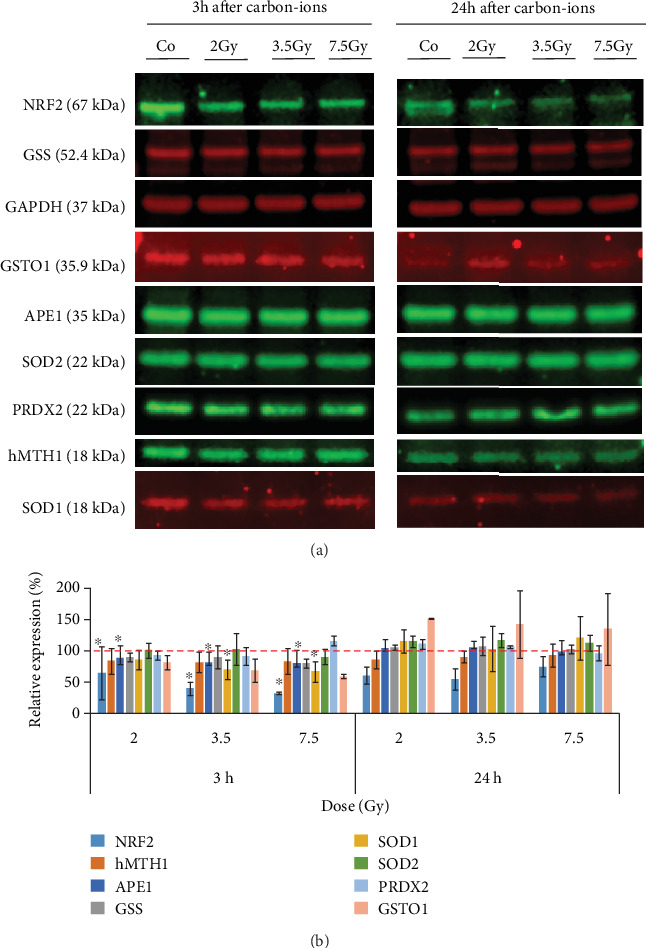
Western Blot analysis of GSS, GSTO1, hMTH1, SOD1, SOD2, APE1, and NRF2 in neurosphere cells irradiated with 2, 3.5 and 7.5 Gy of carbon ions (a). Quantitative analysis of the WB bands was used to calculate the relative expression of each protein normalized by GAPDH (b). ∗*P* ≤ 0.05, *n* = 3.

Carbon ion irradiation also decreased the overall antioxidant protein expression. A significant reduction was found for NRF2 expression after 2 (*P* = 0.027), 3.5 (*P* = 0.05), and 7.5 Gy (*P* = 0.001); APE1 after 2 (*P* = 0.04), 3.5 (*P* = 0.006), and 7.5 Gy (*P* = 0.004); and SOD1 after 3.5 (*P* = 0.006) and 7.5 Gy (*P* = 0.049). After 24 hours, most of the proteins presented expression levels similar to controls (Figures 9(a) and 9(b)).

Regarding the results of protein expression for cells exposed to 3 different radiation qualities, we observed a trend of downregulation for almost all doses (except by 3.5 Gy low dose rate), 3 h after treatment; the expression levels reached the steady-state levels again for carbon ions while for high dose rate gamma irradiated cells, the expression levels remained downregulated (Figures 7–9).

### 3.5. Effects of NRF2 Knockdown on Protein Expression and Survival

After transfecting cells with plasmids CRISPR/Cas9 for *NRF2* during 72 h, cells were seeded at single cell levels in 96-well plates. Thirty transfected clones originated from different single transfected cells were further subcultured. Only eight clones, namely, N1, N2, N3, N5, N8, N11, N15, and N18, could grow and produce appropriate numbers of cells for protein extraction and other analyses.

**Figure 10:**
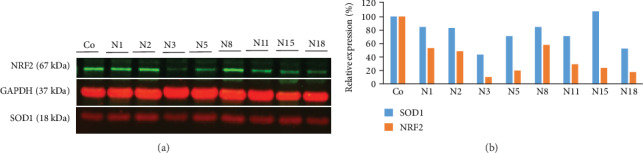
Western Blot analysis of NRF2 and SOD1 in NRF2 knockdown cells of U87MG neurospheres (a). Quantitative analysis of the WB bands was used to calculate the relative expression of each protein normalized by GAPDH (b).

Analysis of NRF2 expression in the transfected clones (NRF2-KD) showed several clones presenting more than 50% NRF2 knockdown. The N3 clone presented the lowest NRF2 expression, but these cells stopped growing after 2-3 weeks. The clone N18 was growing well and was chosen for the study. As a direct target of NRF2, SOD1 protein was also analyzed and showed about 50% decrease in expression levels for several clones, including N18 (Figures 10(a) and 10(b)). The clone N18 was expanded and established as a cell line, and some vials were kept at -140°C for the study.

**Figure 11:**
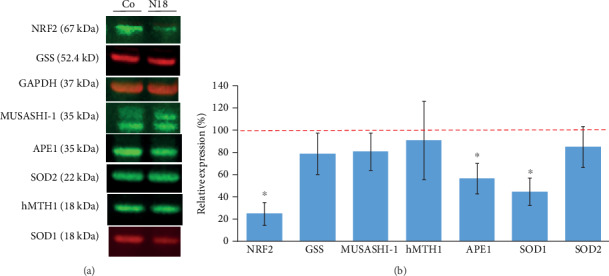
Protein expression analyzed in NRF2 knockdown cells by CRISPR/Cas9 (a). The data were normalized to GAPDH expression. Relative protein expression was calculated by comparing the expression levels of NFR2 knockdown cells with control cells (b). ∗*P* ≤ 0.05.

Expression of NRF2 protein was analyzed in N18 NRF2-KD cells. The average expression of NRF2 was 79% lower (*P* ≤ 0.001) than the expression levels of NRF2 in nontransfected control cells. We also tested the expression of several proteins, e.g., GSS, SOD1, SOD2, hMTH1, APE1, and MUSASHI-1 in the N18 NRF2-KD cells. Interestingly, a significant lower expression was found for APE1 (*P* = 0.003) and SOD1 (*P* ≤ 0.001), with a reduction of 47% and 59%, respectively, compared with controls (Figures 11(a) and 11(b)).

**Figure 12:**
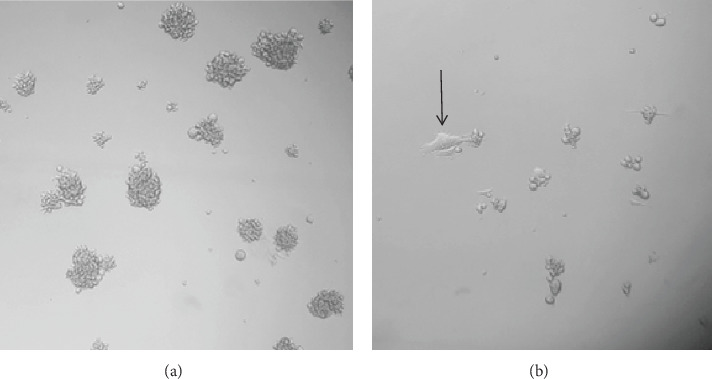
Spheres from control (a) and N18 NRF2-KD (b) formed from 500 seeded cells, after one week at culture conditions. The arrow shows a differentiated cell. 600x magnification.

In fact, we observed the impact of NRF2 knockdown in neurosphere cells, leading to differentiation, decrease in the cell size, and a reduced number of spheres as compared to the control nontransfected neurospheres (Figure 12).

**Figure 13:**
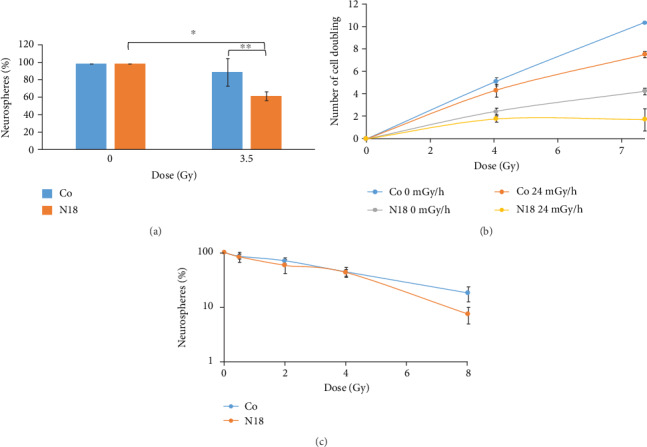
Neurosphere formation (a), and number of GSC NRF2 nontransfected cell doublings (b) in N18 (NRF2-KD) cells after low dose rate gamma irradiation. Formation of neurospheres following the exposure of cells to high dose rate of gamma rays (c).

After characterizing the N18 cell line regarding oxidative cell response-related proteins, we examined cellular sensitivity to low and high dose rate gamma radiation, compared to the control neurospheres with normal expression of NRF2. After 6 days of low dose rate irradiation (24 mGy/h, 3.5 Gy), we observed a significant decrease in the number of N18 neurospheres compared with the sham-irradiated N18 control (*P* = 0.049) and also in comparison with the irradiated nontransfected neurospheres displaying normal expression of NRF2 (*P* ≤ 0.001) (Figure 13(a)). The number of cell doublings was significantly lower in N18 control (nonirradiated NRF2-KD) compared to neurosphere control after 6 days (*P* ≤ 0.001) and 13 days (*P* ≤ 0.001). Low dose-rate irradiation decreased proliferation rate in both neurospheres (at 7.5 Gy, *P* ≤ 0.001) and N18 (3.5 Gy, *P* = 0.041and 7.5 Gy, *P* = 0.035) cells compared to sham-irradiated controls (Figure 13(b)). However, when we use percentage values (relative to control), the reduction observed in cell proliferation was significantly higher in N18 cells compared to neurospheres at 6 days (*P* = 0.004) and 13 days (*P* = 0.022) following low dose rate gamma exposure.

High dose rate gamma irradiation induced a significant decrease in the number of spheres presented by both, control and NRF2-KD neurospheres, following irradiation with 4 Gy (*P* ≤ 0.05). A significantly lower amount of spheres was found in NRF2-KD cells exposed to 8 Gy (*P* = 0.018), compared to NRF2-normal cells exposed to the same dose, Figure 13(c). These results show that NRF2 knockdown increased the sensitivity of neurosphere cells to low dose rate irradiation rather than to high dose rate gamma irradiation.

## 4. Discussion

In the present study, we investigated the responses of neurospheres, at molecular and cellular levels, to low/high dose rates of gamma and carbon ion radiation, and analyzed the consequences of NRF2 knockdown on the radiosensitivity of GBM. Neurospheres are known to be formed by GSCs and their transit-amplifying cells, which indicate tumor heterogeneity in humans [63]. In this work, we showed that the efficiency of neurosphere formation analyzed for U87MG cells was 27% and cells within the neurospheres expressed 34% higher MUSASHI-1 marker compared with differentiated cells, as analyzed by Western Blot. Previously, our group showed a larger number of CD133 positive cells in neurospheres compared with differentiated cells and a neurosphere formation efficiency of 16% [61]. In another article, the authors reported 3.5% of efficiency for U87MG neurospheres [12] and 3 to 5% for primary GBM cells [64]. We also showed a tendency for higher expression of oxidative stress response proteins, e.g., GSS, GSTO1, hMTH1, SOD1, SOD2, APE1, and NRF2 in U87MG neurospheres, compared with differentiated U87, LN18, T98G, M059K, and M059J.

Considering the formation of spheres following irradiation, we found that carbon ion irradiation was more cytotoxic to the neurosphere cells than low and high dose rate gamma irradiation. The LD50 for each of the radiation qualities was 1.3 Gy for carbon ions, 3.5 Gy for high dose rate of gamma, and 11.1 Gy for low dose rate of gamma radiation. Previously, it was shown that charged particles seem to be more effective in GSC killing [18]. This has been suggested to be due to the production of multiple damage sites (MDS), which are extremely cytotoxic and difficult to repair. The advantage of carbon ions in terms of cell killing was already reported in both putative colon cancer stem cells and normal primary fibroblasts compared to photons [65, 66].

The relevance of extracellular 8-oxo-dG induction in irradiated cells was previously shown [57]. By knocking down hMTH1, we have previously shown that the extracellular 8-oxo-dG originates from the reaction between reactive oxygen species and cytoplasmic contents of dGTP [57], which lead to the formation of 8-oxo-dGTP, a mutagenic nucleotide, since during its replication, 8-oxo-dGTP can be incorporated into the DNA and generate transversion due to mispairing with adenine [67]. MTH1 converts 8-oxo-dGTP to 8-oxo-dGMP, which is released from the cells as 8-oxo-dG. Furthermore, we previously showed that extracellular 8-oxo-dG is a sensitive marker of oxidative stress [58, 68]. In this work, we also found a linearly increasing level of extracellular 8-oxo-dG after exposure of neurosphere cells to low dose rate gamma irradiation, while a slight increase was found for carbon ion-irradiated cells, and an absence of this effect was found in high dose rate gamma-irradiated cells.

The results are in accordance with our previous report, where we demonstrated that exposure of cells to low dose rates of gamma radiation led to oxidative stress and increased production of 8-oxo-dGTP, which is released to the medium as 8-oxo-dG. In cells exposed to 0.5 and 1 Gy gamma rays (high dose rate), increased levels of 8-oxo-dG were not observed [69]. Since cellular responses to ionizing radiation depend on DNA damage processing and repair, these processes are likely to be involved in the differential cellular responses regarding radiation qualities. Exposure of cells to doses of 0.5 and 1 Gy at a high dose rate is toxic because DNA damage occurs within a very short time, while low dose rate gamma exposure is less cytotoxic because cells can handle the induced DNA damages as they occur over a prolonged exposure time. Consequently, the damaged irradiated cells are able to effectively deal with DNA damage and also remove modified dNTPs, such as 8-oxo-dGTP, induced by ROS at low dose rate exposure.

However, there is a tendency of increased levels of 8-oxo-dG in carbon ion-irradiated samples compared to high dose rate gamma-irradiated cells (Figure 6(b)). It was previously reported that primary fibroblasts, VH10 cells, presented higher levels of 8-oxo-dG in the media when irradiated with carbon ions, compared to cells exposed to a high dose rate of gamma radiation. In contrast to our results, cells exposed to a high dose rate of gamma also presented higher levels of 8-oxo-dG [40]. This can be due to the different schedules used in each study. In the present investigation, we collected media 3 hours after treatment, while in the other investigation, samples were collected at 24 hours post irradiation [66]. This wave of oxidative stress was kept even 2 weeks after irradiation [66, 70].

Considering the three radiation qualities used, a trend of downregulation of oxidative stress response markers was observed 3 hours after exposure, with the exception of 3.5 Gy for low dose rate radiation, precisely, downregulation of NRF2 for low dose rate (11.5 Gy), high dose rate (7.5 Gy), and carbon ions (2, 3.5, and 7.5 Gy); SOD1 for low dose rate (3.5 Gy), carbon ions (3.5 and 7.5 Gy); hMTH1 for low dose rate (3.5 and 7.5 Gy) and high dose rate (3.5, 7.5 and 11.5 Gy); GSS for high dose rate (3.5, 7.5, and 11.5 Gy) and APE1 for carbon ion (at 2, 3.5, and 7.5 Gy) irradiation. At 24 hours post irradiation, the trend of the protein downregulation remained in cells irradiated with high dose rate gamma radiation, but the expression returned to base-line in cells irradiated with carbon ion. Interestingly, neurospheres exposed to high dose rate gamma radiation showed APE1 (7.5 Gy) and SOD2 (7.5 and 11.5 Gy) expression significantly lower than the nonirradiated control. For instance, the protein expression analysis for low dose rate irradiated cells was performed only at 3 hours post irradiation. The reason was that exposure of cells to low dose rate slowed down the growth rate of the cells (in accordance with our previous report), and when the accumulated doses were reached, the number of cells was too low to perform protein expression analysis at the two post irradiation time points (3 and 24 h).

It is interesting to mention that NRF2 downregulation led to SOD1 downregulation. Similar results were obtained in the CRISPR-Cas9 NRF2-knockdown cells and also in cells (M059K and M059J) that did not express NRF2. However, it is not known whether the decrease in the expression of oxidative stress response proteins is due to lower transcription (lower mRNA levels) of the corresponding genes or by specific protein degradation, considering that the expression levels of the housekeeping protein GAPDH are similar in the control and the irradiated groups. But it can also be due to an adaptation process by expression of genes involved in other protection mechanisms.

While SOD activity in the skin fibroblasts was increased, GPx and catalase activities were not altered immediately after the exposure to photon radiation; the exposure of cells to carbon ion radiation led to lower expression of SOD, GPx, and catalase [42]. These results are partially similar to ours, since we showed lower expression of oxidative stress response proteins. An exception is the increased SOD activity in X-ray-irradiated fibroblasts reported in reference [42], while we observed a downregulation of SOD under all irradiation conditions. In fact, SOD expression was investigated in our study, but we did not evaluate SOD activity; furthermore, it should be taken into account that different experimental designs with different cell lines, cell culture conditions, and time points evaluated might influence the results.

NRF2 regulates basal and inducible expression of enzymes, controlling key components of endogenous antioxidant systems [34]. Considering that low dose rate irradiation induces oxidative damage [69], we knocked down NRF2 under the hypothesis that this might increase the sensitivity of neurospheres to oxidative stress at low dose rate radiation. After knockdown, NRF2 expression remained stable with an average of 79% lower expression than the corresponding NRF2 wild-type cells. This knockdown significantly reduced the expression of SOD1 and APE1. It is interesting to point out that cell lines presenting a very low expression of NRF2, e.g., M059K and M059J, also presented a very low expression of hMTH1, SOD1, SOD2, and APE1. The low expression of NRF2 in M059K and M059J may be related to low APE1 expression, considering that APE1 participates in the NRF2 downregulation through NRF1 activity [71]. While SOD1 and SOD2 have been found to be modulated by NRF2 [28, 29], it is not clear if expressions of APE1 and hMTH1 are related to NRF2 expression.

We also observed that NRF2 knockdown in neurosphere cells caused a decreased expression of the stem cell marker MUSASHI-1, and this may be related to the increase of GSC differentiation. As a consequence, the NRF2 knockdown in neurosphere cells increased the amount of differentiated cells within the spheres, as well as lowered numbers of spheres and decreased rates of neurosphere cell proliferation. The impact of NRF2 knockdown in the differentiation process might be related to higher stress response due to lower expression of SOD1 and APE1. In the literature, it was observed that NRF2 knockdown blocked proliferation of GSCs *in vitro* and *in vivo*, thus reducing the expression of SOX2, BMI-1, and Cyclin E, which are important proteins related to self-renewal, and increasing GFAP, a differentiation marker. Another interesting feature of the NRF2 knockdown was the change of cell shape, very similar to our observation regarding a greater amount of differentiated cells, forming mixed neurospheres or differentiated colonies [72]. Another study also suggested that the downregulation of NRF2 increases GSCs differentiation, by decreasing the sphere-like colonies and also increasing the amount of dendritic cells in the spheres [73]. It was also reported that NRF2 knockout cells showed elevated ROS levels in k-ras-transformed cells, lower proliferation rate, elevated amount of apoptosis *in vitro*, and a reduced tumor growth *in vivo* [74].

Effects of NRF2 knockdown in neurosphere cells also caused a high sensitivity to low dose rate radiation (3.5 Gy) and high dose (8 Gy) of high dose rate gamma radiation, probably due to decreased antioxidant response in these cells. Our results are similar to those obtained by Rocha et al. [75], who observed that the downregulation of NRF2 in serum-cultivated GBM cells increased their sensitivity to TMZ by reducing proliferation, increasing sub-G1 population, and increasing H2AX-positive cells. Furthermore, the authors showed a slow progression of NRF2 knockdown tumor cells independent on TMZ treatment. Knockout of NRF2 in human embryo kidney tumor cells also increased the sensitivity to multiple anticancer agents, including phenethyl isothiocyanate, doxorubicin, etoposide, and cisplatin [74]. A downregulation of SGK1, upstream of NRF2, also increased the effects of radiation and modulated the oxidative stress response in GBM cells [50].

While SGK1 knockdown induces autophagic cytotoxicity in GBM cells, similarly, blocking NRF2 enhances autophagy in untreated and TMZ-treated GBM cells [76]. Considering that the mechanism of action of both treatments includes oxidative damage, autophagy might be an important player in the responses to radiation-induced oxidative damage in GBM neurosphere cells that were submitted to NRF2 knocked down.

**Figure 14:**
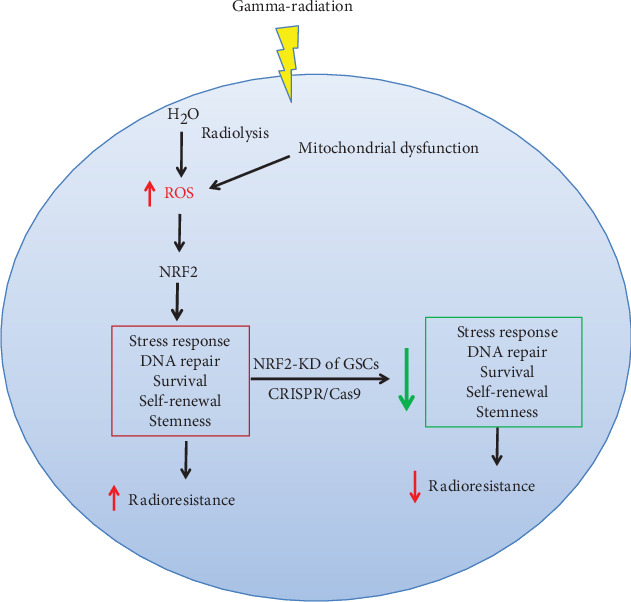
Biological functions attributed to NRF2 are possibly implicated in GSC radioresistance.

Taken together, these results indicate that NRF2 knockdown exerted a great impact in cellular responses to irradiation, by decreasing the antioxidant properties of neurospheres, leading to a lesser self-renewal capacity, and increasing the cell differentiation. Moreover, the increase in cell radiosensitivity reinforces the role of NRF2 as a key regulator of pathways related to oxidative damage responses, thus indicating NRF2 as a molecular target for reducing GBM cell survival (Figure 14).

## 5. Conclusions

Overall, we have shown a picture of how GBM neurosphere cells respond to different conditions, including differentiation status, radiation qualities, and NRF2 gene knockdown. We observed that higher oxidative stress in neurosphere cells reflected a lower proliferation and could be related to the lower expression of most of the analyzed stress response proteins compared to the low dose rate irradiated differentiated cells, including NRF2 protein.

NRF2 knockdown decreased antioxidant properties of neurosphere cells leading to less self-renewal and more differentiation. NRF2 knockdown drastically increased the sensitivity of the neurospheres to the low dose rate irradiation and to a lesser extent to the high dose rate gamma irradiation (only at the highest dose of 8 Gy). Importantly, the increased sensitivity of neurospheres to low dose rate gamma irradiation inducing oxidative stress [49] makes it clear that NRF2 is a good target for reducing GBM cell survival by exposing cells to agents that cause oxidative stress.

## Figures and Tables

**Table 1:** LD50 values (Gy) for each of five cell lines after gamma irradiation.

Cell line	Average ± SD (Gy)
LN18	4.8 ± 2.8
T98G	3.7 ± 0.8
M059J	0.7 ± 0.1
M059K	2.8 ± 0.5
U87MG	4.2 ± 0.7

## Data Availability

The data used to support the findings of this study are included within the article.
